# Spatially Explicit Analysis of Metal Transfer to Biota: Influence of Soil Contamination and Landscape

**DOI:** 10.1371/journal.pone.0020682

**Published:** 2011-05-31

**Authors:** Clémentine Fritsch, Michaël Cœurdassier, Patrick Giraudoux, Francis Raoul, Francis Douay, Dominique Rieffel, Annette de Vaufleury, Renaud Scheifler

**Affiliations:** 1 Department of Chrono-Environment, UMR UFC/CNRS 6249 USC INRA, University of Franche-Comté, Besançon, France; 2 Université Lille Nord de France, Lille, France; 3 Laboratoire Génie Civil et géoEnvironnement (LGCgE), EA 4515, Lille, France; University of Kansas, United States of America

## Abstract

Concepts and developments for a new field in ecotoxicology, referred to as “landscape ecotoxicology,” were proposed in the 1990s; however, to date, few studies have been developed in this emergent field. In fact, there is a strong interest in developing this area, both for renewing the concepts and tools used in ecotoxicology as well as for responding to practical issues, such as risk assessment. The aim of this study was to investigate the spatial heterogeneity of metal bioaccumulation in animals in order to identify the role of spatially explicit factors, such as landscape as well as total and extractable metal concentrations in soils. Over a smelter-impacted area, we studied the accumulation of trace metals (TMs: Cd, Pb and Zn) in invertebrates (the grove snail *Cepaea sp* and the glass snail *Oxychilus draparnaudi)* and vertebrates (the bank vole *Myodes glareolus* and the greater white-toothed shrew *Crocidura russula*). Total and CaCl_2_-extractable concentrations of TMs were measured in soils from woody patches where the animals were captured. TM concentrations in animals exhibited a high spatial heterogeneity. They increased with soil pollution and were better explained by total rather than CaCl_2_-extractable TM concentrations, except in *Cepaea sp*. TM levels in animals and their variations along the pollution gradient were modulated by the landscape, and this influence was species and metal specific. Median soil metal concentrations (predicted by universal kriging) were calculated in buffers of increasing size and were related to bioaccumulation. The spatial scale at which TM concentrations in animals and soils showed the strongest correlations varied between metals, species and landscapes. The potential underlying mechanisms of landscape influence (community functioning, behaviour, etc.) are discussed. Present results highlight the need for the further development of landscape ecotoxicology and multi-scale approaches, which would enhance our understanding of pollutant transfer and effects in ecosystems.

## Introduction

Trace metals (TMs) are naturally present in the environment, however, soils can exhibit high levels of these persistent pollutants due to anthropogenic activities, and such contamination is recognised as a subject of concern for both organism and ecosystem health [1], [2]. Assessment of the environmental and ecological factors that may influence the transfer of pollutants in ecosystems is a key issue in ecotoxicology. However, the understanding of this phenomenon is hampered by the frequently high spatial and temporal variability of soil and landscape factors, which dramatically affects exposure pathways of receptors [3]–[6]. It is recognised that the exposure of organisms to contaminants varies spatially due to heterogeneity in the level of soil contamination, the environmental availability of the pollutant, the habitat and landscape characteristics, and some “host factors” related to ecological and behavioural characteristics of the organism (habitat preferences, home range size, feeding behaviour, migratory behaviour, etc.) [6]–[10]. In heterogeneous landscapes, both the duration and the intensity of exposure may vary spatially because the time spent by the animal (due to foraging behaviour for instance) in the different patches constituting the landscape is likely to change according to land use [11]. Moreover, levels of TMs in soil can also vary between habitats or landscapes because interception and retention characteristics of certain habitats increase TM levels in soil. Additionally, differences in environmental TM availability between land uses may occur due to changes in soil characteristics, soil biodiversity and speciation of metals in soil [6], [12]–[20]. Finally, landscape patterns modulate the structure and the functioning of populations and communities [21], [22] and could therefore be an important factor governing the spatial heterogeneity of organism exposure.

Several authors have emphasised the necessity of studying factors that may affect pollutant bioavailability on the same spatial scale at which harmful effects on receptors occur (i.e., at the habitat or landscape level) [3], [4], [23]–[25]. Some authors have provided evidence for the influence of soil contamination, land use heterogeneity and home range size on organism exposure using modelling methods [10], [26]–[29]. In most cases, these studies developed individual-based random walk models and calculated the cumulative exposure of animals. An effort has also been made to introduce spatially explicit data, such as home range size, contamination heterogeneity and foraging behaviour, in the risk assessment [23], [30]–[32]. Concepts and developments for a new field in ecotoxicology referred to as “landscape ecotoxicology” were proposed in the 1990s [33], but not much research has been developed in this new field. While the influence of spatially explicit ecological variables on exposure has been explored with modelling, the effective differences in the exposure of organisms between land uses or landscapes of a specific site have rarely been assessed in the field [19]. To our knowledge, only one recent study has investigated the influence of habitat type on TM bioaccumulation, and it was found that the transfer of pollutants from soil to biota varied among habitats [19].

Land snails and small mammals have been extensively studied for use as biomonitors of TM environmental contamination due to their bioaccumulation abilities, which have shown that TM levels in tissues (referred to as “internal” levels hereafter) reflected the contamination level of sites [34]–[37]. The relationships between internal TM levels in animals and in soils have mainly been studied by comparing polluted sites to reference areas, which does not allow investigating the influence of the spatial heterogeneity of total or available soil TMs on bioaccumulation. By studying the accumulation of metals in small mammals from diffusely polluted flood plains, Wijnhoven et al. [38] investigated the relationship between TM concentrations in animals and soils sampled at the same locations. These authors found weak correlations between body and soil TM concentrations and suggested that dispersal of animals and changes in foraging and feeding behaviour due to periodic flooding influenced their exposure to contaminants. Conversely, in a recent study on TM accumulation in small mammals along a pollution gradient in a smelter-impacted area, we observed that internal TM levels were highly dependent upon local soil contamination [7]. While studying TM accumulation in wood mice, van den Brink et al. [39] showed intersite differences in soil-kidney Cd ratios, which they attributed to soil parameters that drive soil TM availability to plants and invertebrates. Such discrepancies highlight the potential influence of site-specific characteristics (for instance landscape, environmental particularities such as flooding or barriers, and soil properties) on TM transfer in food webs, and highlight the need for data on the influence of environmental characteristics on pollutant transfer in ecosystems, considering both the intensity and the spatial pattern of transfer.

Metal transfer from soil to biota is controlled in large part by TM bioavailability, which may be defined as the fraction of the total concentration of a metal that is available or can be made available for uptake and, as a consequence, for causing effects in organisms [8], [40]–[42]. TM availability is often estimated using chemical extractants; among these, calcium chloride (CaCl_2_) is considered to be relevant for the assessment of the fraction of cadmium (Cd), lead (Pb) and zinc (Zn) that is bioavailable to some plants and invertebrates [40], [42]–[45]. Because plants and soil invertebrates may constitute the dietary items for a large variety of invertebrate and vertebrate consumers, it could be hypothesized that CaCl_2_-extracts of TMs reflect their potential to be transferred in food webs. It is commonly admitted that organisms respond (accumulation and/or effects) to bioavailable rather than total metal concentrations in soils, and from a risk assessment perspective, it has been recommended that studies be developed based on bioavailable rather than total TM concentrations for risk estimates [45]. In the present work, we studied four sympatric species in a large polluted site surrounding a former Pb and Zn smelter. These species differed based on their phylogeny (invertebrates versus vertebrates), physiology (TM storage abilities), diet (herbivorous, omnivorous, and carnivorous), spatial mobility and habitat preferences. Small mammals have a larger spatial range of mobility than snails [10], [46]–[50]. The grove snail *Cepaea sp* is an herbivorous species [51], [52] while the glass snail *Oxychilus draparnaudi* is carnivorous [53]–[55]. The bank vole *Myodes* (ex-*Clethrionomys*) *glareolus* is herbivorous/granivorous and intermediary between strictly herbivorous voles and omnivorous mice [47], [56], [57], while the greater white-toothed shrew *Crocidura russula* is typically carnivorous [48]. We addressed three questions: (i) Are TM concentrations in animals better related to total or chemically-extractable soil TM concentrations? (ii) Does the landscape influence the relationship between TM concentrations in animals and in soils? (iii) At which spatial scale do TM concentrations in animals correlate best with soil TM concentrations? The latter question relies upon the hypothesis that the correlation will be strongest when the spatial range considered for soil contamination will approximate the surfaces exploited by the organism of concern.

## Materials and Methods

### Study site and sampling strategy

This study was conducted in the surrounding area of the former “Metaleurop-Nord” smelter in Northern France (Noyelles-Godault, Nord – Pas-de-Calais, 50°25′42 N, 3°00′55 E). The contamination of agricultural and urban soils around Metaleurop has been well documented [58]–[61], and soils of woody habitats were also recently studied [20], [62], thus showing that the area is highly polluted by Cd, Pb and Zn for both levels of contamination and surfaces of concern.

We defined a 40 km^2^ (8×5 km) study area, which was divided into 160 squares (500×500 m), that constituted our sampling units. In each square, soils and animals were sampled in woody habitats. Habitats classified as “woody” consisted of natural forests, tree plantations (e.g., poplar groves), woodlots or copses and hedgerows in natural or cultivated lands and urban parks. Of the 160 squares, nine were excluded from the sampling; one was excluded because it corresponds to the location of the former smelter, which was undergoing rehabilitation process and the others because they were occupied by ploughed fields only and thus lacked any woody habitats. The number of squares actually sampled was therefore 151.

### Landscape analysis

A land use analysis was performed to determine the landscape composition of each square. For this purpose, the study area was extended to 9×6 km by adding a line of squares around the initial 8×5 km grid with the aim of avoiding an edge effect in further statistical analyses. Land use mapping was accomplished using ArcGIS 8 (ESRI Co., USA) on the basis of the CORINE Land Cover (CLC) database (European Commission, 2000). The resolution of CLC is 25 ha. Based on aerial photographs (BD ORTHO® database from the *Institut Géographique National,* resolution of 0.5 m) and on field reconnaissance when necessary, the limits of the different units and the units smaller than 25 ha (such as hedgerows or copses) were manually digitalised in order to obtain a resolution of four meters. The resulting vector map was converted into a raster map (1 pixel = 4×4 m), which was composed of eight categories of land use: urban and industrial, ploughed field, short grass, shrub and tall grass, hedgerow and copse, forest, river and pond, and former Metaleurop Nord smelter. For each square, the number of pixels of each category of land use was computed. Seven groups of squares were identified using correspondence analysis and clustering, and these groups corresponded to seven landscape types. These types (hereafter referred to as “landscape”) were named according to their land use matrix: agricultural lands, urban areas, woodlands, shrublands, mixed urban areas and agricultural lands, and mixed woodlands and grasslands. The last type was constituted of the former Metaleurop smelter itself. The four prevailing landscape types were agricultural lands, urban areas, woodlands and shrublands. These four landscape types were used to answer the second question of this work, which addressed the influence of landscape on the relationship between TM concentrations in soils and in individuals of the different species studied.

### Soil use analysis

To answer the third question of this study, which addressed the scale at which TM concentrations in soils and animals correlate best, we needed to obtain soil contamination data in buffers of increasing radius size. To achieve this aim with the best accuracy, our data on “woody” soils were gathered along with data on agricultural and urban soils that were obtained from the soil database of the *Equipe Sols et Environnement* (LGCgE, Groupe ISA, Lille, France).

The land use map was used to build a soil use map with four types of soil use: woody soils (soils of woody patches), agricultural soils (soils of ploughed fields and grasslands) and urban soils (soils of urban areas such as gardens and parks). Dredged sediment deposits, which are extremely contaminated and do not technically

### Soil, small mammal and snail sampling

In each of the 151 squares, one to 10 composite soil samples were obtained from woody patches during the autumn of 2006 [20], [62]; this sample number varied depending on logistical issues, such as the number of available woody patches and the accessibility of the patches. Each composite sample in a woody patch comprised 15 randomly placed elementary samplings, and these 15 sub-samples represented the same weight part in the composite sample. The first 25 cm of soil were sampled, and the litter layer (OL layer, which is a layer on the soil surface that accumulates little to no decomposed leaves and woody fragments) was removed. However, the humus layer (OF layer, which consisted of fragmented residues) was sampled with the top mineral soil material, consistent with the most frequently recommended protocol in Europe. A total of 262 soil samples were analysed over the 40 km^2^ study area. Detailed data regarding soil physico-chemical parameters and contamination are published elsewhere [20], [62].

The sampling of animals, which could not have been performed on all 160 squares for logistical reasons, was conducted on 30 squares. These 30 squares were chosen in order to obtain three replicates from three levels of pollution (low, medium and high) in the four prevailing landscape types defined above (agricultural lands, urban areas, woodlands and shrublands). Because the landscape type “shrublands” was present only in the vicinity of the former smelter, we obtained only one level of pollution (high) for this type of landscape. Small mammals were captured during the autumn of 2006 using break-back traps baited with a mix of water, flour and peanut butter. Sampling authorisation was obtained from the DIrection Régionale de l'ENvironnement (DIREN) of Nord – Pas-de-Calais. In each square, 10 lines of 10 traps, each spaced three meters apart, were placed throughout the woody patches where the soils were sampled. In three squares, the available surfaces of woody patches were insufficient to place 10 lines of traps; the number of lines was therefore reduced to six or seven. The 289 trap lines were checked every morning for three consecutive days and were re-set/rebaited as necessary. The sampling effort consisted of 2820, 600, 2310 and 2940 trap-nights in agricultural lands, shrublands, urban areas and woodlands, respectively (one trap set for one night corresponds to a “trap-night”). The percentage of captures was calculated as the number of individuals trapped per 100 trap-nights. Snails were collected by hand searching in the same woody patches and at the same time as small mammals. Snails and small mammals were stored at −20°C until dissection. Each capture location (trap line for small mammals, woody patch for snails) was georeferenced using a GPS (Garmin eTrex®).

### Animal preparation

Animals were identified at the species level using morphometric criteria [47], [54], [63], [64] and were dissected. Crystalline lens weighing is a standard method for estimating the relative age of small mammals and is relevant for the bank vole [65]. However, it was not used here because a significant proportion (10%) of the crystalline lenses of bank voles was not usable because they were broken by trapping and/or freezing. Moreover, to our knowledge, no validation of this age determination method has been published for the greater white-toothed shrew. Therefore, we decided to classify the small mammals (shrews as well as voles) into three classes of relative age (juveniles, non-reproductive adults and reproductive adults) on the basis of body size, body weight and reproductive status [7]. We found significant differences in crystalline weight between these three classes, suggesting that our age classification was acceptable.

Small mammals were dissected to sample the liver, which was dried in a 60°C oven to achieve a constant dry weight before acid digestion and TM analyses (see next paragraph). Snails of the *Cepaea* genus (*C. hortensis* and *C. nemoralis*) were grouped and will be referred to hereafter as *Cepaea sp* because specific determination is not possible for juveniles. Furthermore, preliminary analyses of our data showed that TM accumulation did not differ between adult *C. hortensis* and *C. nemoralis*. *Cepaea sp* snails were classified according to two classes of relative age (juveniles and adults) based on the presence of a clear lip at the mouth of their shell. The presence of this lip indicates that the snail has attained adulthood [66]. The age of *Oxychilus draparnaudi* specimens could not be determined because no published method was available. The soft bodies of the snails were separated from the shells and dried in a 60°C oven to achieve a constant dry weight before TM analysis. Three to five individuals of *Oxychilus draparnaudi* were combined to achieve a sufficient sample weight for TM analysis.

### Analyses of TM concentrations in soils and animals

Woody soils were analysed for total and CaCl_2_-extractable metal concentrations. Samples were dried, disaggregated and homogenised before being sifted using a 250 µm mesh. Cd and Pb concentrations were measured with inductively-coupled argon plasma mass spectrometry (ICP-MS), and Zn concentrations were measured with inductively-coupled argon plasma atomic emission spectrometry (ICP-AES) after acid digestion according to the NF X31-147 standard procedure [67]. Measurements were performed by the *Laboratoire d'Analyse des Sols* of the *Institut National de la Recherche Agronomique* (INRA) of Arras (France), which benefits from the COFRAC accreditation n°1–1380 for the analytical quality of its TM measurements in soils. All precautions were taken with respect to protocol application and calibration; quality control was achieved using procedural blanks, Certified Reference Materials (CRM, namely: BCR 141 and 142; GBW 07401, 07402, 07404, 07405 and 07406), samples from inter-laboratory comparisons, internal control samples and duplicates of the analysis. Selective extractions using CaCl_2_ (0.01 M) were conducted in triplicate on 3 g sub-samples of soil. Extractable metal concentrations were quantified using atomic absorption spectrometry (AAS, AA-6800, Shimadzu) by the *Equipe Sols et Environnement* (LGCgE, Groupe ISA, Lille, France). Quality control was performed on these measurements, using procedural blanks and CRM (BCR 483). The average recoveries of the CRM varied between 90% and 110%.

Metal concentrations in snail soft bodies and small mammal livers were measured using furnace (Cd, Pb) or flame (Zn) atomic absorption spectrometry (VARIAN 220Z and 220FS, respectively) at the Chrono-Environment department (UMR 6249 UFC/CNRS, Besançon, France). Digestion of samples was performed by dissolution in nitric acid (HNO_3_, 65%, Carlo Erba analytical quality) in a dry oven (65°C) for 72 h. After digestion, samples were diluted by the addition of ultra-pure water (18.2 MΩ/cm^2^). Blanks (acid + ultra-pure water) and CRM (TORT-2 and DOLT-3, National Research Council, Canada) were prepared and analysed using the same methods as the samples. Average recoveries of the CRM were calculated at 95%±10% (*n* = 38) for Cd, 101%±17% (*n* = 42) for Pb, 80%±2% (*n* = 22) for Zn. Detection limits of the spectrometers (median±3 SD of blanks) were 0.17, 1.2 and 2.8 µg.l^−1^ in the acid digests for Cd, Pb and Zn, respectively. Detection limits in snail soft bodies were 0.02, 0.12 and 0.29 µg.g^−1^ in the grove snail and 0.09, 0.65 and 1.5 µg.g^−1^ in the glass snail for Cd, Pb and Zn, respectively. Detection limits in small mammal livers were 0.02, 0.13 and 0.30 µg.g^−1^ in the bank vole and 0.03, 0.24 and 0.56 µg.g^−1^ in the greater white-toothed shrew for Cd, Pb and Zn, respectively. When measured values were under the detection limits, half of the detection limit value was used for statistical analyses. For both soil and animal samples, metal concentrations were expressed as microgram per gram dry weight (µg.g^−1^ DW). Hereafter, body and hepatic TM concentrations of snails and small mammals will be referred to as “internal TM concentrations”.

### Prediction of TM concentrations in soils

To determine at which spatial scale the correlation between TM concentrations in animals and in soils was the best, a circular buffer technique was used in order to calculate soil metal concentration in the area surrounding the animal capture location. Several buffer radiuses were used: 50, 75, 100, 175, 250, 350, 500, 750 and 1000 meters around the sampling point. For each snail or small mammal, at each buffer size, median predicted Cd, Pb and Zn concentrations in the soil were computed. For this purpose, metal concentrations in soils were predicted (universal kriging) throughout the whole 54 km^2^ study area at each node of a regular (100 m) grid. Detailed information about this procedure is given in Supporting Information files (Text S1, Tables S1 and S2, Figure S1).

### Statistical analyses of TM concentrations in animals

For each sampled individual, the following data were available: internal TM concentration (i.e., TM concentration in the liver for the mammals and in the whole soft body for the snails), total and CaCl_2_-extractable TM concentration in the soil of the patch where the individual as was caught (referred to hereafter as TM concentration “at sampling point”), species, age (except for *Oxychilus draparnaudi*), landscape where the animal was found (i.e., landscape type of the square, as defined in the section “Landscape analysis”) and median predicted total Cd, Pb and Zn concentrations in soils at several buffer sizes (as defined in the previous section). The normality of the data distribution was checked with the test of Shapiro-Wilk. Because metal concentrations in soils and animals were skewed, variables were log-transformed for statistical analyses using log_10_(*x*+1). The analysis of the data was divided into three steps to address the three aims of this paper. Statistical analyses were performed using general linear models (LMs, [68]).

The purpose of the first step was to test whether animal and soil TM concentrations at the sampling point were related and whether total or CaCl_2_-extractable soil TM concentrations best explained TM concentrations in animals. As age can influence TM accumulation in small mammals and snails [7], [34], [69], [70], we modelled the relationship between internal and soil TM concentrations controlling for age; residuals of the internal concentration/age relationship (which will be referred to hereafter as “internal concentration normalized to age”) were modelled against total or CaCl_2_-extractable TM concentrations in soils. On the basis of the R^2^ of these models, we determined which soil TM concentration (total or CaCl_2_-extractable) better explained the internal TM concentrations. Subsequently, the best variable was kept for further analyses. We analysed the differences in accumulation between species using LMs with internal metal as the dependent variable and total soil TM concentrations and species with interactions as explanatory variables. Comparisons of accumulation level between species were therefore performed conditionally to total TM concentrations in soils (i.e., placing the variable “total soil TM concentration” first in the model).

In the second step, we considered the influence of landscape on internal TM concentration by adding the variable “landscape” with interaction into the models. Comparisons of accumulation level between landscape types were therefore performed conditionally to age and total soil TM concentrations (i.e., placing these variables first in the model).

Finally, we determined at which spatial scale the animal and soil TM concentrations were most strongly correlated. We hypothesized that this correlation will be strongest when the buffer size approximates the surface exploited by the organism of concern. For this purpose, LMs were used with internal TM concentrations normalized to age as the dependent variable and median predicted total soil TM concentrations in buffers around sampled animals as independent variables. For each species, the R^2^ of the models and the values of the coefficients (slopes) were computed for each buffer size, both on the whole dataset and by landscape. Because the sample size was small for the shrew in woodlands (*n* = 4), the grove snail in urban areas (*n* = 3) and null for snails in shrublands (no specimens), these landscape types were not analysed individually for these species. For each buffer radius size, we recorded the highest and lowest of the calculated partial R^2^ values for soil concentrations and computed the differences between the values, which will be referred to hereafter as “Δ R^2^”. If the value of Δ R^2^ was lower than 0.05, we assumed that the R^2^ did not show straightforward variations with buffer size increase.

n all cases, the significance of the variables was checked via permutation test (Monte-Carlo, 1000 permutations), the partial R^2^ values were calculated using an analysis of variance (ANOVA). Pairwise differences were determined using the Tukey's post-hoc multiple comparison test (Tukey's “Honest Significant Difference” method).

All statistical analyses were performed using the software R 2.7.1 [71] with the following additional packages: ade4, geoR, gstat, maptools, pgirmess, pvclust, spdep, splancs, and vegan.

## Results

**Figure 1:**
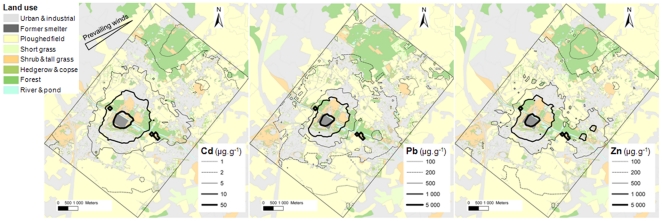
Iso-concentration lines of predicted total Cd, Pb and Zn concentrations in topsoils from Metaleurop-impacted area. Concentrations are expressed as µg.g^−1^ DW.

Total soil TM concentrations varied from background levels to very high values, particularly in the dredged material deposit (Table 1, Table S1). CaCl_2_-extractable TM concentrations also varied widely within woody soils (Table 1). Spatial distribution and predictions of TM concentrations in soils showed a similar spatial pattern for the three TMs (Text S1, Figure S1, Table S2). Concentric circles were found around the former smelter with an enhancement of the contamination in downwind areas (Figure 1). Hot spots corresponding to dredged sediment deposits were also found within the study area (Figure 1).

**Table 1:** Total and CaCl_2_-extractable trace metal concentrations in soils of woody habitats from Metaleurop-impacted area.

Metal	Min.	Med.	Max.	DSD
Cd	0.10	5.0	236	2 402
Pb	16	303	7 331	41 960
Zn	44	460	7 264	38 760
Cd-CaCl_2_	0.02	0.20	5.8	59
Pb-CaCl_2_	0.27	0.66	14	1.7
Zn-CaCl_2_	0.02	4.1	143	112

Min.: minimum, Med.: median, Max.: maximum. Concentrations in woody soils (*n* = 261) and dredged sediment deposit (DSD, *n* = 1). Concentrations expressed as µg.g^−1^ DW.

A total of 131 grove snails (*Cepaea sp.*), 78 glass snails (*Oxychilus draparnaudi*), 248 bank voles (*Myodes ex-Clethrionomys glareolus*) and 163 greater white-toothed shrews (*Crocidura russula*) were captured (Table 2). Even if these four species were present over the whole area, the species were not evenly distributed among landscapes and pollution levels (Table 2). Neither of the two snail species selected were found in the shrubland landscape. Grove snails were abundant in both agricultural and woodland landscapes but rare in urban areas. The glass snail was often present in urban areas but less often in woodlands, and it was rarely found in agricultural areas. The bank vole was clearly more abundant in woodlands than in the three other landscapes, where it was roughly similar in abundance. Greater white-toothed shrews were equally abundant in agricultural and urban areas and were rare in both shrubland and woodland landscapes.

**Table 2:** Distribution of specimens by species, age and landscape and percentage of capture for small mammals.

Species	Age	Landscape				Total
		Agricultural lands	Shrublands	Urban areas	Woodlands	
*Cepaea sp*	Juv	9	0	2	19	30
	Ad	43	0	1	57	101
	Total	52	0	3	76	131
						
*Oxychilus draparnaudi*	Total	6	0	50	22	78
						
*Myodes glareolus*	Juv	7	10	1	54	72
	NR ad	2	7	9	44	62
	Ad	8	4	4	98	114
	Total	17	21	14	196	248
	PC	0.6	3.5	0.6	6.7	2.9
						
*Crocidura russula*	Juv	10	0	13	0	23
	NR ad	50	9	56	4	119
	Ad	15	0	6	0	21
	Total	75	9	75	4	163
	PC	2.7	1.5	3.2	0.1	1.9

Juv: juveniles, NR ad: non-reproductive adults and Ad: reproductive adults, PC: number of individuals trapped per 100 trap-nights.

### Influence of species, age and TMs in soils at the sampling point on internal TM concentrations

Considering all the species together, internal concentrations showed a large range of variation; they varied from under detection limits for Cd and Pb and 0.88 µg.g^−1^ DW for Zn to 741, 443 and 6619 for Cd in the greater white-toothed shrew, Pb in the grove snail and Zn in the glass snail, respectively (Table 3).

**Table 3:** Trace metal concentrations measured in snails (soft body) and small mammals (liver) from Metaleurop-impacted area.

Species	*n*	Cd			Pb			Zn		
		Min	Med	Max	Min	Med	Max	Min	Med	Max
*Cepaea sp*	131	UDL	42	170	1.0	23	443	0.88	447	1 927
*Oxychilus draparnaudi*	78	UDL	55	258	10	55	313	206	91	6 619
*Myodes glareolus*	243	0.13	11	116	0.08	1.8	200	19	89	173
*Crocidura russula*	163	3.0	72	741	UDL	8.8	159	65	139	271

Min.: minimum, Med.: median, Max.: maximum, UDL: under detection limit. Concentrations expressed as µg.g^−1^ DW.

**Figure 2:**
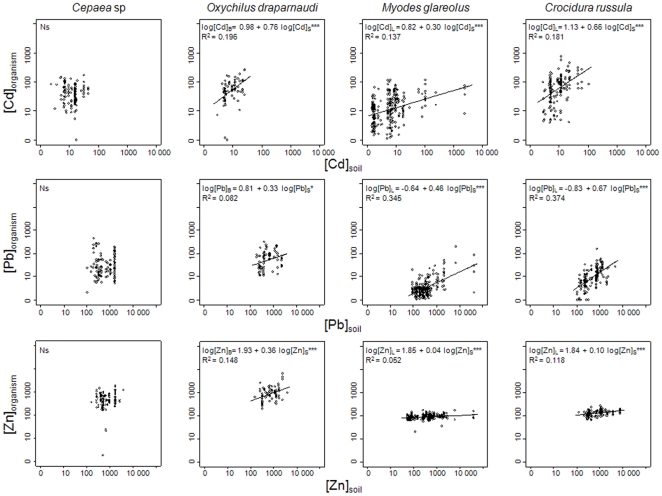
Relationships between Cd, Pb and Zn concentrations in organisms and in soils. TM concentrations in the soft body for snails ([C]_B_) and in the liver for small mammals ([C]_L_), total TM concentrations measured in soil at the sampling point ([C]_s_) (µg.g^−1^ DW). Statistical significance: general linear model, Ns for p>0.05, * for p<0.05, ** for p<0.01, *** for p<0.001, R^2^: R-squared value of the model.

Internal Cd concentrations, conditionally to soil total concentrations (i.e., placing the variable “total soil TM concentration” first in the model), ranked in the following order: greater white-toothed shrew ∼ glass snail > bank vole > grove snail (*p*<0.001, Figure 2). Regarding Pb, internal concentrations, conditionally to total soil concentrations, were higher in small mammals than in snails and ranked in the following order: greater white-toothed shrew > bank vole ∼ glass snail > grove snail (*p*<0.001), while for Zn the rankings were as follows: glass snail > grove snail > greater white-toothed shrew ∼ bank vole (*p*<0.001).

Hepatic Cd concentrations slightly increased with age in small mammals (*p*<0.001, R^2^ = 0.07 for the vole; *p* = 0.002, R^2^ = 0.06 for the shrew, Table 4). Similarly, adult grove snails exhibited higher levels than juveniles (*p*<0.001, R^2^ = 0.22). Pb concentrations, however, did not increase with age in small mammals although they differed with age in the grove snail, with juveniles exhibiting slightly higher concentrations than adults (*p* = 0.045, R^2^ = 0.03). Zn concentrations weakly increased with age in the bank vole (*p*<0.001, R^2^ = 0.03) but did not vary for the shrew or for the grove snail.

**Table 4:** Multiple and partial R-squared for significant parameters of models linking internal TMs with studied variables.

Species	[TM]_internal_	Partial R^2^				R^2^ model
		Age	log[TM]_soil_	Landscape	[TM]_soil_*Landscape	
*Cepaea sp*	log[Cd]	0.22			0.08	0.30
	log[Pb]	0.03		0.16		0.21
	log[Zn]					ns*
						
*Oxychilus draparnaudi*	log[Cd]		0.20		0.09	0.29
	log[Pb]		0.08	0.12	0.12	0.33
	log[Zn]		0.15	0.06	0.17	0.38
						
*Myodes glareolus*	log[Cd]	0.07	0.19	0.11	0.02	0.39
	log[Pb]		0.35	0.08	0.02	0.46
	log[Zn]	0.03	0.08	0.07		0.18
						
*Crocidura russula*	log[Cd]	0.06	0.17	0.07		0.31
	log[Pb]		0.37	0.09	0.03	0.50
	log[Zn]		0.12	0.05		0.19

R^2^ model: multiple R-squared, Partial R^2^: partial R-squared, [TM]_internal_: TM concentrations in animals, [TM]_soil_: TM concentrations in soils at sampling location, [TM]_soil_*Landscape: interaction between TM concentrations in soils and landscape, significance of parameters: *p-value*<0.05.

*ns: non significant *p-value*>0.05.

Internal TM concentrations increased with total soil concentrations at the sampling points in the glass snail and the small mammals, although the grove snail did not show any increase in internal concentrations along the pollution gradient, regardless of the metal considered (Figure 2). Total TM concentrations explained 7 to 37% of the variation in internal concentrations (Table 5). Internal concentrations were always better correlated with total rather than CaCl_2_-extractable TMs (Table 5), except for the grove snail, for which internal concentrations were not related to either total or extractable TM concentrations (except for Cd-CaCl_2_). Internal concentrations were not correlated with extractable TMs in the glass snail. Similarly, Zn in the bank vole and Pb in the greater white-toothed shrew were not significantly related to Zn-CaCl_2_ and Pb-CaCl_2_, respectively.

**Table 5:** Regressions between TM concentrations in animals and in soils (total and CaCl_2_-extractable) at sampling location.

Species	log[TM]_internal_∼log[TM]_soil_		*b*	*p*	R^2^
*Cepaea sp*	Cd	Total	−0.06	0.571	
		CaCl_2_	0.65	0.002	0.07
	Pb	Total	0.06	0.590	
		CaCl_2_	0.13	0.866	
	Zn	Total	0.19	0.070	
		CaCl_2_	0.12	0.059	
					
*Oxychilus draparnaudi*	Cd	Total	0.76	<0.001	0.20
		CaCl_2_	0.74	0.090	
	Pb	Total	0.33	0.011	0.08
		CaCl_2_	0.48	0.275	
	Zn	Total	0.36	<0.001	0.15
		CaCl_2_	0.05	0.430	
					
*Myodes glareolus*	Cd	Total	0.34	<0.001	0.19
		CaCl_2_	0.37	<0.001	0.05
	Pb	Total	0.45	<0.001	0.33
		CaCl_2_	−0.19	0.016	0.02
	Zn	Total	0.05	<0.001	0.07
		CaCl_2_	−0.02	0.121	
					
*Crocidura russula*	Cd	Total	0.64	<0.001	0.18
		CaCl_2_	0.85	0.015	0.04
	Pb	Total	0.67	<0.001	0.37
		CaCl_2_	0.08	0.886	
	Zn	Total	0.10	<0.001	0.12
		CaCl_2_	0.05	0.037	0.03

*b*:coefficient of the regression, *p* value: statistical significance, R^2^: partial R-squared. TM concentrations measured in soft body for snails and liver for small mammals, normalized to age except for *O. draparnaudi*.

### Influence of landscape on internal TM concentrations

**Figure 3:**
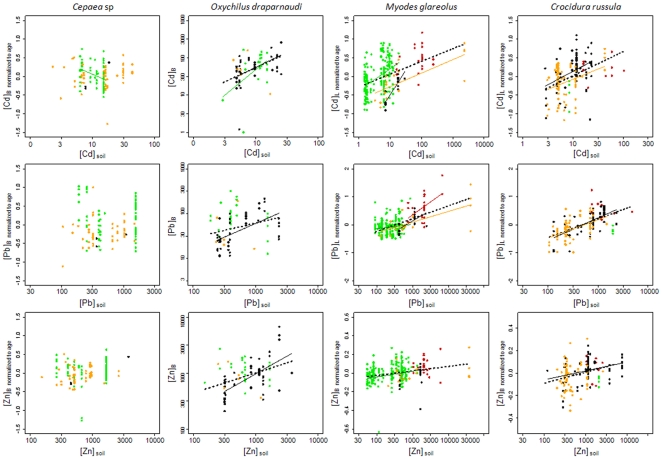
Relationships between TM concentrations in organisms (normalized to age) and in soils by landscape type. TM concentrations in the soft body for snails ([C]_B_) and in the liver for small mammals ([C]_L_) normalized to age (except for *O. draparnaudi*) and total TM concentrations measured in soil at the sampling point ([C]_soil_) (µg.g^−1^ DW). Points are coloured according to the type of landscape where animals were captured (agricultural lands: orange, urban areas: black, shrublands: red, woodlands: green). Significant regressions (*p*<0.05) are plotted with coloured lines that correspond to the landscape of concern, and black dashed lines symbolise the regression for the whole dataset including all landscape types.

Accumulation of metals differed between landscapes for both the levels of internal TM (internal concentration normalized to age and conditionally to total soil TM concentration at the sampling point) and the evolution of internal TM concentrations along the soil pollution gradient, i.e., the slopes of the regressions between internal TM concentrations (normalized to age) and total soil TM concentrations at the sampling points (Table 6, Figure 3). The variable “landscape” accounted for 5 to 16% of the variation in internal TM concentrations, and the interaction between soil contamination and landscape was sometimes significant, particularly for the glass snail (Table 4).

**Table 6:** Parameters of significant regressions between TM concentrations in animals and in soils (total at sampling point) by landscape.

Species	Landscape	*n*	Cd			Pb			Zn		
			*b*	*p*	R^2^	*b*	*p*	R^2^	*b*	*p*	R^2^
*Cepaea sp*	All	131									
	A	52									
	S	0									
	U	3									
	W	76	−0.72	0.002	0.15						
*Oxychilus draparnaudi*	All	78	0.76	<0.001	0.20	0.33	0.010	0.08	0.36	<0.001	0.15
	A	6									
	S	0									
	U	50	0.81	<0.001	0.31	0.61	<0.001	0.34	0.63	<0.001	0.40
	W	22	1.37	0.047	0.23						
*Myodes glareolus*	All	243	0.34	<0.001	0.19	0.45	<0.001	0.33	0.05	<0.001	0.08
	A	16	0.36	<0.001	0.58	0.38	0.007	0.47			
	S	21				0.91	0.028	0.24			
	U	14	1.31	<0.001	0.78	0.76	0.015	0.40			
	W	192	0.58	<0.001	0.19	0.22	0.018	0.04	0.07	<0.001	0.08
*Crocidura russula*	All	163	0.64	<0.001	0.18	0.67	<0.001	0.37	0.10	<0.001	0.12
	A	75	0.54	0.012	0.10	0.65	<0.001	0.24			
	S	9									
	U	75	0.71	<0.001	0.17	0.79	<0.001	0.42	0.08	0.001	0.11
	W	4									

*b*:coefficient of the regression, *p* value: statistical significance, R^2^: partial R-squared. TM concentrations measured in soft body for snails and liver for small mammals, normalized to age except for *O. draparnaudi*. Type of landscape: “A” for agricultural lands, “U” for urban areas, “S” for shrublands and “W” for woodlands.

For *Cepaea* snails in urban areas, the sample size was insufficient and did not allow for reliable analysis. Internal Pb levels were higher in woodlands than in agricultural lands (*p*<0.001), while internal Cd and Zn levels did not differ among landscapes (Figure 3). Internal TM concentrations did not vary with soil TM concentrations, except in the case of Cd in woodlands where a negative correlation was revealed (Table 6, Figure 3).

For sample size reasons, the influence of landscape on TM accumulation in *O. draparnaudi* could only be studied by comparing urban areas and woodlands. Internal Cd and Zn levels did not differ between landscapes, while internal Pb levels were higher in woodlands than in urban areas (*p* = 0.003, Figure 3). Internal Cd concentrations increased with soil TM concentrations in both urban areas and woodlands (without significant differences in slopes between landscapes), whereas internal Pb and Zn concentrations significantly increased along the pollution gradient in urban areas only (Table 6, Figure 3).

For the greater white-toothed shrew, too few animals were collected on woodland and shrubland landscapes and thus no interpretation could be made. However, data from agricultural lands and urban areas showed that the level of internal Cd was higher in urban than in agricultural landscapes (*p* = 0.014, Figure 3), while the slopes of accumulation along the pollution gradient did not differ significantly (Table 6). There were no differences for Pb and Zn in either the level of accumulation or in the increase of internal concentrations along the gradient (Figure 3).

Concerning the bank vole, we found higher levels of internal TMs in shrublands and woodlands compared to agricultural lands and urban areas for Cd (*p*<0.004) and urban areas for Zn (*p*<0.003, Figure 3). Internal Pb levels were higher in shrublands than in other landscape types (*p*<0.01). We did not detect a correlation between internal and soil Cd concentrations in shrublands, while regressions were significant in other landscapes (Table 6, Figure 3). Although the increase in internal Cd concentrations with soil contamination was highest in urban areas and lowest in agricultural lands, the coefficients did not differ significantly between landscapes (Table 6). In contrast, we found a higher increase in internal Pb along the soil pollution gradient in shrublands compared to woodlands and agricultural areas (*p*<0.040, Table 6, Figure 3). The relationships between internal and soil Zn concentrations were rarely significant and did not exhibit differences in slopes between landscapes (Figure 3).

Different patterns could be noticed between non-essential (Cd, Pb) and essential (Zn) metals in small mammals. First, most of the regressions were significant for non-essential metals, in contrast to what was observed for Zn. Second, the increase in internal concentrations along the pollution gradient was higher for non-essential metals (the lowest regression coefficient was 0.22 for Cd and Pb whereas the highest for Zn was 0.10). Finally, inter-individual variability was lower for Zn.

Several patterns linking internal and soil TM concentrations existed, showing various combinations of relatively high/low internal TM levels with sharp/slight increases in internal TMs along the pollution gradient. The relationships between internal and soil TM concentrations varied among landscapes, but these differences were metal-specific and species-specific, thus hampering any generalization (Figure 3, Tables 4 and 6).

The partial R^2^ for the landscape variable was found to be somewhat less important than soil contamination but as important as age for explaining internal TM concentrations (Table 4). The models gathering all the variables (total soil TM concentration, age (except for glass snail) and landscape) explained up to 39% of the variance of the dataset for Cd, 50% for Pb and 38% for Zn (Table 4). Therefore, if the studied variables significantly accounted for internal TM concentrations in snails and small mammals, a part of the inter-individual variability remained unexplained.

### Spatial range of correlation between animal and soil TM concentrations

**Figure 4:**
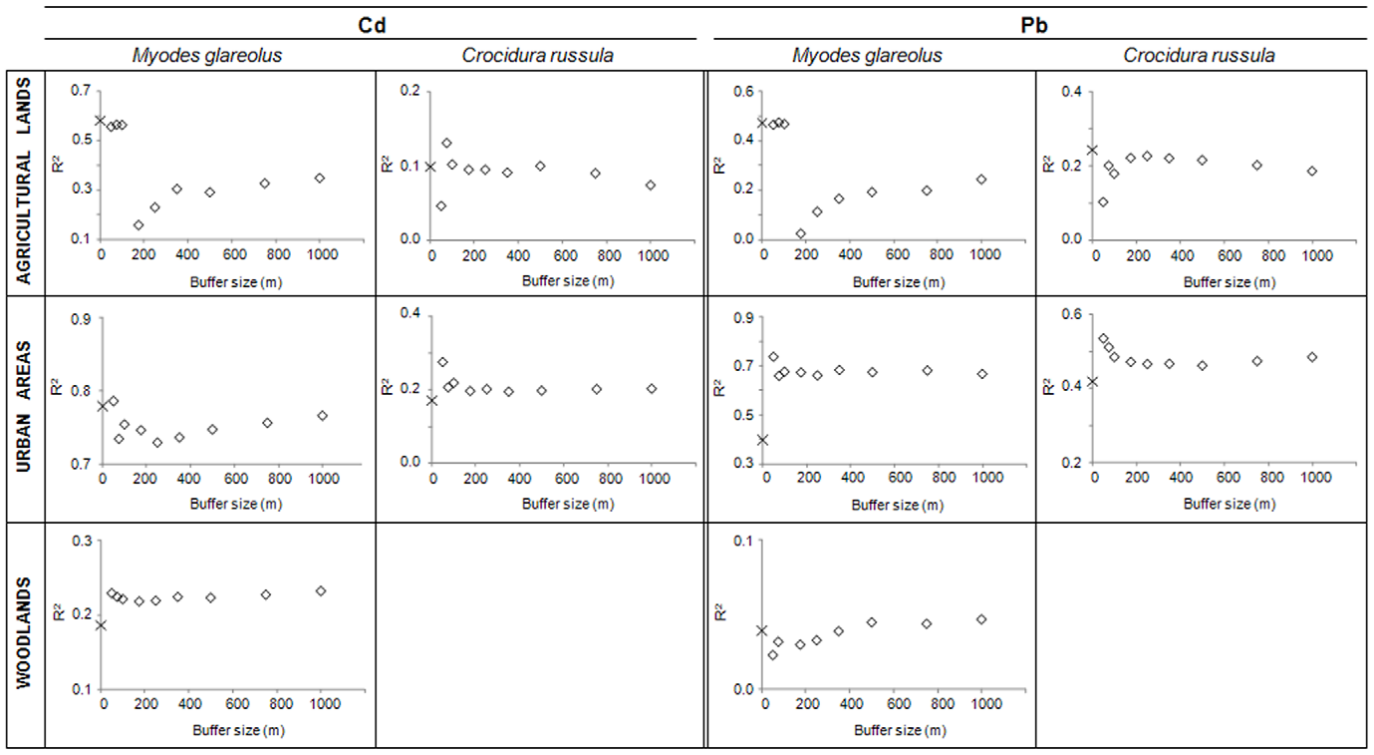
R^2^ values of regressions between TMs in small mammals and in soils at several buffer sizes. Partial R^2^ values for soil Cd and Pb concentrations in significant regressions between TM concentrations (normalized to age) in small mammals and total soil TM concentrations using measured values at the sampling point (×) and predicted values at several buffer sizes (⋄) in agricultural lands, urban areas and woodlands.

**Figure 5:**
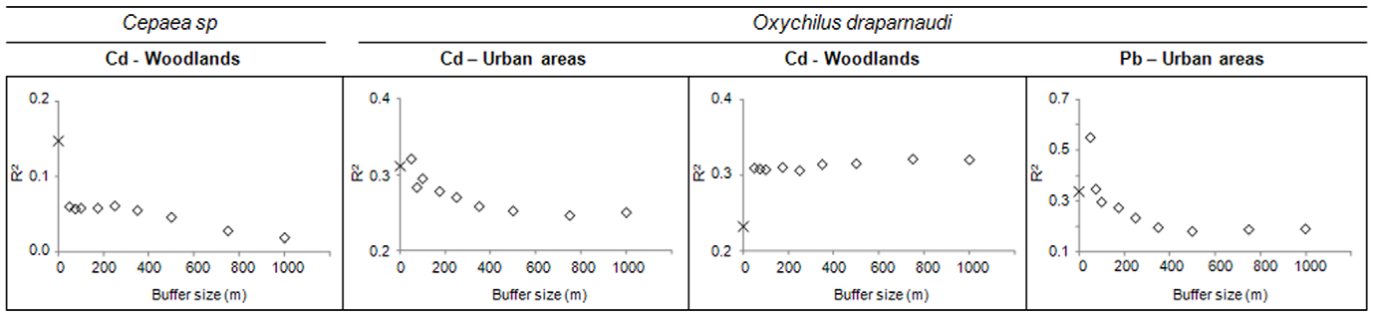
R^2^ values of regressions between TMs in snails and in soils at several buffer sizes. Partial R^2^ values for soil Cd and Pb concentrations in significant regressions between TM concentrations in snails (normalized to age for *Cepaea*) and soil TM concentrations using measured values at the sampling point (×) and predicted values at several buffer sizes (⋄) in urban areas and woodlands.

Globally, TM concentrations in animals were related to soil TM concentrations in buffers, except for the grove snail (for which few relations were significant) and for Zn, which also showed few significant regressions. The number of significant relationships slightly increased from 27 when considering concentrations in soil at the sampling point (Table 6) to 31 (data not shown) when considering concentrations in soil at different buffer sizes. Figures 4 and 5 show the R^2^ values of the regressions between internal concentrations (normalized to age) and concentrations in soils at sampling points, as well as the evolution of the R^2^ values of the models, taking into account the TM concentration in soils at different buffer sizes.

We found four major patterns in the relationships between internal TM concentrations and soil TM concentrations in buffers (Figures 4 and 5). The first pattern is represented by better correlations between internal TM and soil TM concentrations at the sampling point or in buffers of small sizes (<100 m) compared to buffers of larger sizes. This pattern is illustrated by the case of Cd and Pb in bank voles from agricultural lands. A second pattern showed better correlations at the lowest buffer size (50 m) than at the sampling point, followed by a decrease in R^2^ with increasing buffer size. Notably, this pattern can be seen in urban areas, for Cd and Pb in the bank vole, the shrew and the glass snail. However, in this case, the R^2^ values for Cd in bank voles and glass snails were almost equal using TMs at the sampling point and in the 50 m buffer. A third pattern exhibited a better correlation using TM concentrations in buffers rather than at the sampling point, with the R^2^ value remaining stable regardless of the buffer size. This is the case for Cd in the bank vole and in the glass snail from woodlands. Finally, a fourth pattern showed the lowest R^2^ at a 50 m buffer size, but exhibited roughly similar R^2^ values for TMs at the sampling point and in buffers from 75 to 1000 m. This pattern can be seen for Cd and Pb in shrews from agricultural lands. For the grove snail, calculations could be performed for Cd in woodlands only, and showed that internal concentrations were better correlated using Cd concentrations in soils at the sampling point rather than in buffers, albeit with a negative correlation.

As for the landscape effect, spatial patterns and correlations between animal and soil TM concentrations were found to be metal-dependent, species-dependent and landscape-dependent.

## Discussion

### Relationships between internal and soil TM concentrations

The extensive, high-level TM contamination in soils surrounding the former Metaleurop smelter was reflected by the high metal levels measured in sampled organisms, which appeared among the highest reported in the literature for the various species studied [34], [36], [38], [69], [70], [72]–[75].

In contrast to the study of Notten et al. [74] on *Cepaea* snails in the Biesbosch floodplain, we did not observe an increase of TMs in soft bodies with total soil TMs, which could be due to a seasonal effect [70]. Differences in bioaccumulation between seasons may be due to several factors, most notably to variations in snail physiology, snail behaviour and/or contamination of their diet. Our study was conducted in autumn while the study of Notten et al. [74] was performed during the summer, and indeed, *Cepaea* snails sampled in the field or exposed in microcosms in the surroundings of Metaleurop during springtime exhibited an increase of internal TM concentrations along the pollution gradient (unpublished data and [76]). Previous field and laboratory studies have also shown that internal TM concentrations in *Cepaea* snails were better related to diet than to soil TM concentrations [74], [77]. The only significant relationship between TM concentrations in *Cepaea* snails and in soils was observed for CaCl_2_-extractable Cd. This could be due to the fact that CaCl_2_, which is usually a good extractant for the estimation of phytoavailable Cd concentrations [43], [44], may have estimated the concentration of their vegetal diet relatively well. This was not the case for the glass snail, which has a carnivorous diet and had internal TM concentrations that were only related to total soil TMs. *Cepaea* snails have been shown to excrete both Pb and Zn [70], [76]. Their ability to excrete these metals may explain the lack of a relationship between internal and soil concentrations. *Oxychilus* snails exhibited a stronger increase of internal Zn along the pollution gradient compared to *Cepaea* snails, suggesting the existence of different Zn regulation strategies among these taxa.

Recent research has been devoted to the study of the accumulation of TMs in small mammals in polluted sites, taking into account both total and extractable TM concentrations in soils In contrast to our results, in a study in Dutch floodplains, no correlation between internal Cd, Pb or Zn in and total concentrations in soils were found for both the bank vole or the greater white-toothed shrew [38], while correlations between internal Cd and soil Cd-CaCl_2_ for the greater white-toothed shrew and between internal Zn and soil Zn-CaCl_2_ for the bank vole were observed. In the same environmental context of the Dutch floodplains, van Gestel [41] found that soil invertebrates and small mammals showed increases in levels of Cd (and sometimes Cu and Pb) while soil pore water and CaCl_2_ extracts of metals were low, highlighting that available soil metal concentrations were not suitable indicators of metal accumulation in the food chain. In a smelter-impacted site, Rogival et al. [78] used ammonium nitrate as chemical extractant and showed significant relationships between Cd and Pb concentrations in organs of the wood mouse and in its diet (acorns and earthworms). They found stronger relationships between the diet and soil TMs using total rather than extractable concentrations for several metals including Cd, Pb and Zn. Thus, if total concentrations in soils are better estimates of the contamination of the diet than extractable ones, this may explain why we found here better correlations between internal TMs and soil TMs using total rather than extractable concentrations. The slight increase in hepatic Zn concentrations in small mammals along the pollution gradient is consistent with previous studies, which have provided evidence for slight differences between polluted and control field sites and the ability of small mammals to regulate internal levels of this essential element [7], [34], [69], [79], [80].

The use of chemical extractants to estimate bioavailability of metals is strongly dependent on the site, the metal, and the species under consideration, rendering any generalization risky. Based on our results and the discrepancies observed in the literature, it seems, as it has been demonstrated for invertebrates and vegetation [43], [81]–[83], that the relevance of chemical extractants for assessing the bioavailability of metals in field situations is also a great matter of concern for vertebrate wildlife. The availability of TMs in soils may not be the main factor governing TM transfer in food webs because bioavailability to herbivorous, omnivorous and carnivorous organisms cannot be predicted by a single chemical extract from a polluted soil. This phenomenon is probably due to the variety of exposure routes [6], [84], [85] and to the fact that factors affecting transfer to secondary consumers are not only related to the partitioning of metals in soils, but also to the parameters that modulate bioavailability along food webs, notably the levels and the chemical storage forms of TM in dietary items as well as the availability of these items [86], [87].

### Influence of landscape on animal TM concentrations

Our results show that landscape composition represents a significant variable that, together with the soil TM concentration and the age of the animals, contribute to explain internal TM concentrations in snails and small mammals. The density, availability and diversity of dietary items are likely to vary among landscape types and it has been proven, for both snails and small mammals, that diet varies according to the availability of dietary items [48], [51], [57], [88]. The feeding and foraging behaviour of animals can therefore change according to the landscape type. Resulting variations in diet composition could affect the amount and the bioavailability of metals transferred along food webs, leading to variable levels of bioaccumulation in the organism considered. Indeed, it has been shown that TM bioavailability for an organism depends not only on its own digestive characteristics but also on the amount and the sequestration of metals within the food [87], [89]–[92]. It has been emphasised that metal bioavailability can be affected by foraging and feeding behaviour of the organism [6], [8], [93]. The different patterns of Cd and Pb accumulation between landscapes found in the present work could thus be partially related to such food chain effects. This was also suggested by Hendrickx et al. [86], who observed site-specific TM accumulation in invertebrates (spiders and amphipods) and hypothesized that biological characteristics of sites, via alterations of trophic webs, can modify TM transfer.

Using individual-based models, Schipper et al. [10] modelled the influence of environmental heterogeneity in Dutch floodplains (soil contamination, habitat availability and suitability) on metal exposure using four species of small mammals, including the bank vole. These authors concluded that environmental heterogeneity governed only a minor part of the variation in metal exposure and that intra-species differences in exposure should mainly be due to inter-individual variations in species traits. In our study, soil contamination and landscape explained a non-negligible amount (around 30%) of the variation in internal Cd concentration. This could be due to the fact that we examined a larger pollution range and we considered not only the land use of the sampling point, but the influence of the landscape (a complex mosaic of land uses) surrounding the sampling point. The examination of exposure heterogeneity by combining these two scales of perception (i.e., heterogeneity within and between landscapes) could improve the understanding of ecological factors affecting transfer of TMs in ecosystems. Multi-scale approaches have been shown to be relevant within the context of biological contaminant (e.g. parasite) transfer in eco-epidemiology [94]–[96] and also appear promising for studies on chemical transfer within a landscape ecotoxicology framework.

Our data revealed a high inter-individual variability in TM concentrations, and although we considered age, soil TMs and landscape, more than half of the variance remained unexplained. Therefore, our results are also partly in accordance with those of Schipper et al. [10] concerning the strong influence of intra-species variability on exposure and TM accumulation. This phenomenon suggests that individual characteristics and behaviour govern a large part of the variations in internal concentrations.

### Spatial scale of correlation between animal and soil TM concentrations

Because internal and soil TM concentrations at the sampling point scale were correlated, we argue that the present study provides evidence for a spatially explicit relationship between TM concentrations in animals and in soils. Our results stress that this spatial correlation is due to both the levels of metals in soils and the landscape composition around the habitats where the animals were sampled. The improvement of the correlation between internal and external concentrations using an increasing buffer size was not straightforward, in contrast to what was expected. Marinussen and van der Zee (1996) modelled exposure to and accumulation of Cd in fictitious organisms with various sizes of home-range (10 to 400 m^2^) and showed that home-range size greatly affected exposure [26]. We assume that the range of the strongest correlation between internal and soil metal concentrations depends on numerous biological and ecological parameters. In fact, accumulation abilities, inter-individual variability, spatio-temporal variations in exposure, and heterogeneity in levels of soil contamination may affect the strength of the relationship between accumulated and soil TM concentrations.

### Overall interpretation of the results with the example of Pb

**Figure 6:**
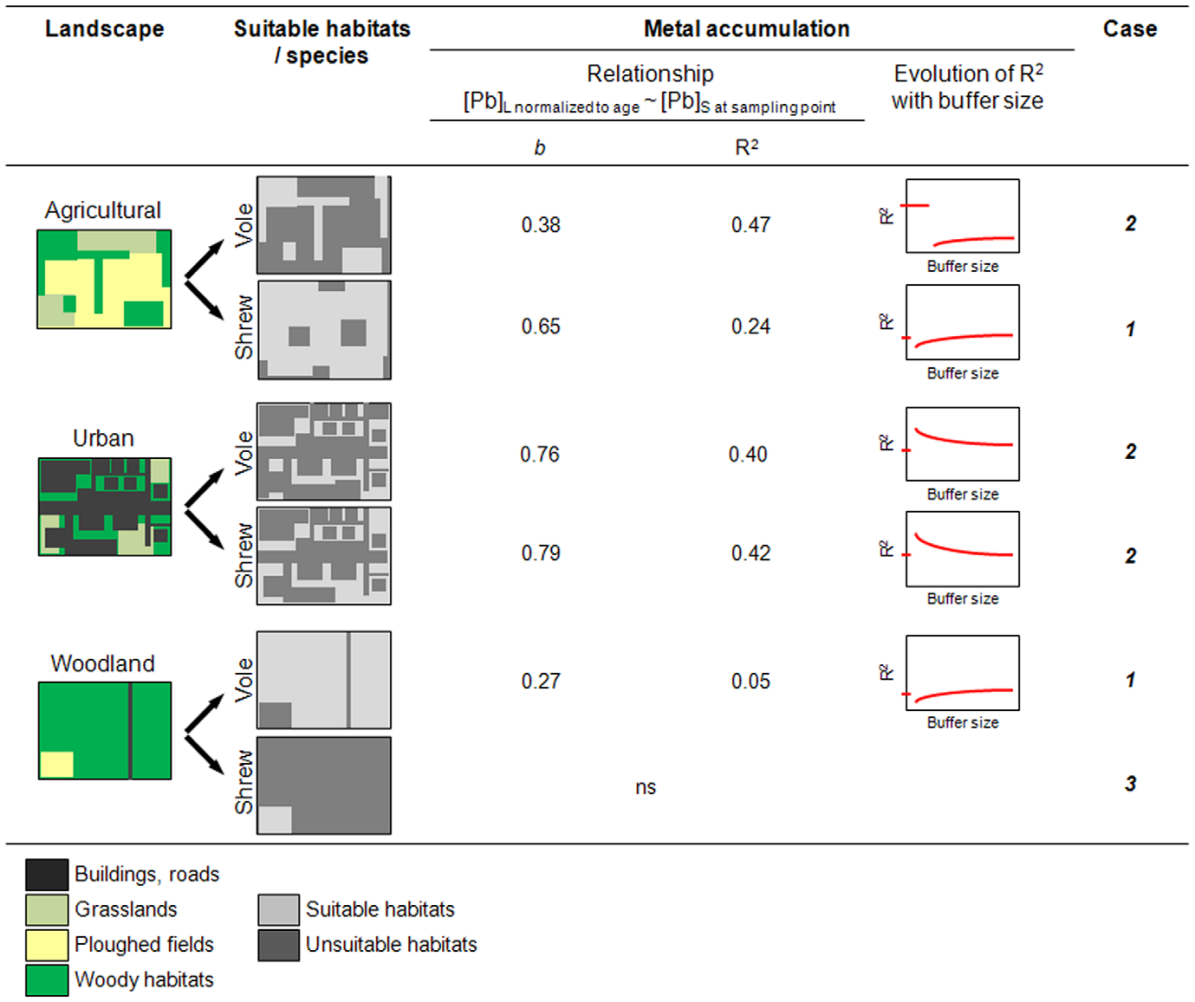
Summary and overall interpretation of the data with the example of Pb in small mammals. The data concerning Pb in the bank vole and in the greater white-toothed shrew is summarised, thus illustrating our overall interpretation of the data. Data concerning metal accumulation are R^2^ values and slopes of the regressions between Pb concentrations in the liver (normalized to age) and total Pb concentrations in soil measured at the sampling point in addition to patterns of evolution of the R^2^ values with buffer size. Maps of landscapes are based upon observed situations within the study area. Maps of suitable habitats for each species are hypothesized on the basis of habitat preferences described in the literature. Three patterns linking internal TM concentrations to soil TM concentrations have been uncovered, these patterns are named “Case 1, 2 and 3”.

The movements of small mammals are known to be variable among landscapes because of the spatial heterogeneity of suitable habitats, their connectivity and the characteristics of the ecological barriers [49], [97]. Presence and survival of animals in heterogeneous landscapes are related to both site-specific characteristics, which define habitat quality on a local scale, and to factors acting at the landscape and/or meta-population scale [98]. Landscape features, animal habitat preferences and resource requirements govern the spatial repartition of suitable/unsuitable patches over an area [22]. This can result in different exposure patterns among landscapes for a given species, and can lead to different inter-species exposure within the same landscape type [4], [25]. Within this context, we propose a synthetic interpretation of the results we obtained from small mammals, using Pb as an example (Figure 6). We hypothesize that relationships between internal and soil TMs are modulated by both landscape and the ecological characteristics of the species. In other words, we propose that the exposure of each species and the resulting bioaccumulation are dependent upon its ecological characteristics (such as spatial behaviour and diet) which are likely to be landscape-specific.

We have built hypothetical maps of suitable and unsuitable habitats on the basis of habitat preference data found in the literature. The bank vole is a forest specialist species. It can live in other woody habitats (for instance woodlots and hedgerows) in heterogeneous landscapes (agricultural, urban or others) where it is confined to woody patches and rarely moves to surrounding habitats unless it needs to cross them to reach other suitable woody habitats [49], [99]–[101]. The greater white-toothed shrew is more eurytopic. This species is mostly present in inhabited areas and open landscapes such as agricultural fields [48], [97], [101], [102]. Therefore, within each landscape, habitat suitability differs between voles and shrews.

We propose the existence of three different cases (Figure 6). When almost the entire area is suitable (case 1), home-ranges can overlap several habitats that differ in metal contamination and individuals can easily move between more or less contaminated patches. Therefore, internal concentrations show high inter-individual variability and are poorly related to soil contamination, as evidenced by the relatively low R^2^ values and slopes of the relationships). Moreover, the strength of the relationship between internal and soil TMs does not substantially vary regardless the considered scale for soil TM concentration (the R^2^ value was roughly similar between the sampling point and buffers as well as among buffers). This is the case for shrews in agricultural lands and voles in woodlands. Conversely, when animals are confined to suitable isolated patches within a hostile matrix (case 2), the absence or the presence of high local contamination in occupied patches would greatly condition animal exposure, since individuals cannot easily move or forage in surrounding habitats. Internal concentrations therefore would more strongly depend on soil TM concentration in occupied patches, resulting in better correlations between internal and soil contamination compared to case 1. In this case, represented by voles in agricultural lands and voles and shrews in urban areas, we found globally strong relationships between TMs in animals and in soils (high R^2^ values and/or slopes). Moreover, we observed better correlations on local scales (at the sampling point or at a buffer size lower than 100 m). Finally, in landscapes characterised in a large part by unsuitable habitats (case 3), animals can be found during migration and dispersal or in margins, and it may be assumed that the main duration of exposure has occurred in another place. In such cases (represented by shrews in woodlands and shrublands), we captured few individuals, all of which were non-reproductive adults, and we failed to detect relationships between TMs in animals and in soils.

We observed that landscape composition influences bioaccumulation with species-specific patterns. Thus we conclude that landscape features modulate animal exposure to pollutants, and this effect might differ according to species ecological characteristics. The spatial heterogeneity of soil contamination could more or less affect exposure, depending on the species and the landscape concerned.

### Conclusions

Apart from animal age and metal concentration in soil at the sampling point, the landscape around the habitat of capture influences the internal TM concentrations. Certain landscapes are therefore more at risk than others depending on the considered organisms and metals. In some cases, transfer is high even at low levels of soil contamination and result in elevated accumulated TM levels in animals (i.e., an elevated biota to soil accumulation factor). In other cases, low internal TM levels were found at low soil TM concentrations, but internal concentrations sharply increased along the pollution gradient. Both phenomena were also found to co-occur. We propose that a landscape gathering both phenomena would the most at risk for wildlife.

The lack of relationship between internal levels in animals and CaCl_2_-extractable TM concentrations in soil, as well as the differences in relationships between animal and soil TM concentrations between studies, provide two major insights within a risk assessment framework. First, the use of chemical extracts may not be relevant when assessing TM bioavailability to herbivorous/granivorous and carnivorous species. Second, predictions of internal TM using general accumulation models, such as the use of global regression regardless of the environmental characteristics of a site (e.g. landscape) and the intra-species variability should be used cautiously.

Exposure of wildlife to contaminants and subsequent TM accumulation is determined by several parameters acting at different biological organisation levels that are integrative variables of several processes. Based on our results, we suggest that TM accumulation in snails and small mammals is governed by ecological (diet, habitat preferences, mobility, etc) and physiological (assimilation and excretion of TM) characteristics of animals. Our results strongly suggest that availability in soil does not fully determine transfer in food webs. Species ecology and landscape are other key factors that determine organism exposure. Our findings lead us to hypothesize that ecological characteristics, such as food web structure and the way that organisms exploit their environment (home-range size, migration, feeding behaviour, habitat preferences, etc), both dependent on landscape features, mainly explain TM transfer in food webs. The present study points out the need for further investigation to develop a field of “landscape ecotoxicology” and elucidate the underlying mechanisms behind landscape effects, and additionally highlights the interest for a multi-scale approach in ecotoxicology.

## Supporting Information

Figure S1 **Empirical omnidirectional variograms with variographic envelopes and retained fitted models for Cd, Pb and Zn.**
(TIF)

Table S1 **Total concentrations of trace metals in soils from Metaleurop-impacted area, according to soil use type.**
(DOC)

Table S2 **Parameters of the fitted variogram models, results of cross-validation and median variance of kriged values.**
(DOC)

Text S1 **Prediction of trace metal (Cd, Pb, Zn) concentrations in topsoils of Metaleurop-impacted area.**
(DOC)
