# Supplementary figures and images for: Spatially Explicit Analysis of Metal Transfer to Biota: Influence of Soil Contamination and Landscape
Source: PLoS One. 2011 May 31;6(5):e20682. doi: 10.1371/journal.pone.0020682 (PMC3105103; doi:10.1371/journal.pone.0020682)

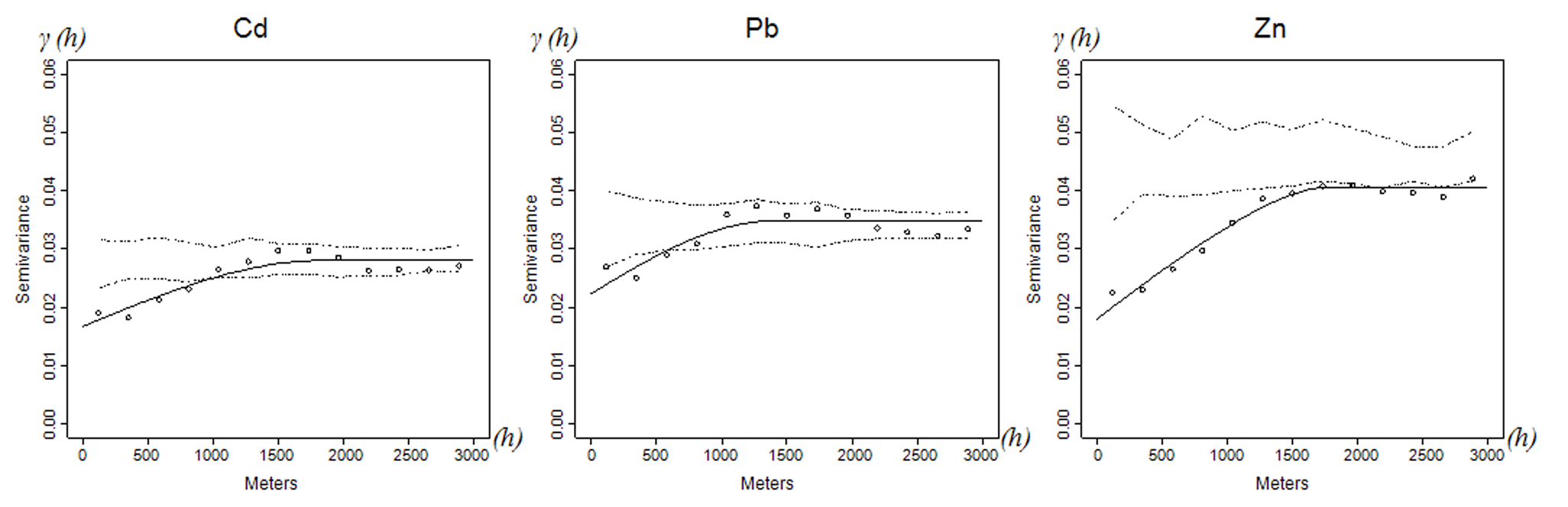

Supplement: Figure S1 — Empirical omnidirectional variograms with variographic envelopes and retained fitted models for Cd, Pb and Zn. (TIF) [file pone.0020682.s001.tif]
