# Supplementary material for: Spatially Explicit Analysis of Metal Transfer to Biota: Influence of Soil Contamination and Landscape
Source: PLoS One. 2011 May 31;6(5):e20682. doi: 10.1371/journal.pone.0020682 (PMC3105103; doi:10.1371/journal.pone.0020682)
Supplement: Table S1 — Total concentrations of trace metals in soils from Metaleurop-impacted area, according to soil use type. (DOC) [file pone.0020682.s002.doc]

| Metal | Soil use | Minimum | Median | Maximum |
| --- | --- | --- | --- | --- |
| Cd | A | 1.6 | 5.1 | 44 |
|  | U | 2.5 | 12 | 31 |
|  | W | 0.10 | 5.0 | 236 |
|  | D |  | 2 402 |  |
|  |  |  |  |  |
| Pb | A | 80 | 265 | 3 005 |
|  | U | 124 | 794 | 3 711 |
|  | W | 16 | 303 | 7 331 |
|  | D |  | 41 960 |  |
|  |  |  |  |  |
| Zn | A | 124 | 376 | 2 515 |
|  | U | 203 | 858 | 5 830 |
|  | W | 44 | 460 | 7 264 |
|  | D |  | 38 760 |  |

Concentrations expressed as µg.g^-1^DW. Soil use: “A” for agricultural (*n* = 294), “U” for urban (*n* = 39), “W” for woody (*n* = 261) soils and “D” for dredged material deposits (*n* = 1).
