# Supplementary material for: Spatially Explicit Analysis of Metal Transfer to Biota: Influence of Soil Contamination and Landscape
Source: PLoS One. 2011 May 31;6(5):e20682. doi: 10.1371/journal.pone.0020682 (PMC3105103; doi:10.1371/journal.pone.0020682)
Supplement: Table S2 — Parameters of the fitted variogram models, results of cross-validation and median variance of kriged values. (DOC) [file pone.0020682.s003.doc]

| Metal |  | Variogram model | | | |  | Cross-validation | | | | |  | Kriging |
| --- | --- | --- | --- | --- | --- | --- | --- | --- | --- | --- | --- | --- | --- |
|  |  | Correlation function | Nugget | Partial sill | Range |  | ME | MSPE | MSDR | *r* observed and predicted | *r* predicted and residuals |  | Median variance kriged values |
| Cd |  | Spherical | 0.017 | 0.011 | 1846 |  | <0.001 | 0.03 | 1.40 | 0.84 | -0.02 |  | 0.05 |
| Pb |  | Spherical | 0.022 | 0.012 | 1385 |  | <0.001 | 0.04 | 1.48 | 0.82 | -0.02 |  | 0.07 |
| Zn |  | Circular | 0.018 | 0.022 | 1706 |  | <0.001 | 0.04 | 1.65 | 0.81 | -0.02 |  | 0.06 |

Range expressed as meters. ME: mean error, MSPE: mean square predicted error, MSDR: mean squared deviation ratio, *r* : Pearson product-moment correlation coefficient.
