# Supplementary material for: Spatially Explicit Analysis of Metal Transfer to Biota: Influence of Soil Contamination and Landscape
Source: PLoS One. 2011 May 31;6(5):e20682. doi: 10.1371/journal.pone.0020682 (PMC3105103; doi:10.1371/journal.pone.0020682)
Supplement: Text S1 — Prediction of trace metal (Cd, Pb, Zn) concentrations in topsoils of Metaleurop-impacted area. (DOC) [file pone.0020682.s004.doc]

Data available from the present study and the from the database of the *Equipe Sols et Environnement* (LGCgE, Groupe ISA, Lille, France) for the prediction of total trace metal (TM) concentrations in soils concerned 294, 39 and 262 sampling points for agricultural, urban and woody soils, respectively (Table S1). Among these last 262 data, the substrate of one sampled point corresponded to a dredged sediment deposit and thus was not a soil in the strict sense. This sediment came from the Deûle River, which has received effluents and wastes from Metaleurop smelter for decades and was extremely polluted. This point was therefore considered separately from the other woody soils to avoid overestimating TM concentrations in woody habitats surrounding this particular site. Moreover, it was used to interpolate TM concentrations in a similar site, which also received dredged sediments from the Deûle but was not sampled in this work.

Previous studies conducted in the surroundings of the former Metaleurop Nord smelter have reported that Cd, Pb and Zn concentrations in soils decrease with the distance from the smelter [1-3]. Furthermore, it has been shown that winds may influence rates of atmospheric deposition of contaminated dusts, and thus influence soil pollution, with an enhancement of contamination at downwind locations [4-8]. Metal levels can also depend on land use as evidenced by several studies: woodlands are generally more contaminated than open lands (grasslands, arable lands, moorlands or heathlands) due to higher deposition and retention [9-14]. Moreover, in urban areas, some of the variability in metal levels is related to land use because of differences in the source of contamination and in deposits [15-17]. Consequently, we studied the relationships between metal concentrations in soils and the distance to Metaleurop, the frequency of wind and the soil use (agricultural, urban, sediment deposits, and woody).

The Euclidean distance (expressed in meters) between each soil sampling point and the source (centre of the former Metaleurop plant) was calculated. The distance between sampling points and Metaleurop varied from 472 to 5795 m. Wind data were obtained from Météo-France (Paris, France). The dataset consisted of 18 238 values, which were daily measurements of instantaneous wind at a height of 10 m from January 1958 to December 2007 at the Lille-Lesquin meteorological station (approximately 20 km away from Metaleurop). A compass rose, which integrated wind direction and frequency (%), was obtained using these data. The frequency of the wind blowing was reported for each class of angle on the compass rose circle (from 0 to 360°, with a step of 20°), and the sum of the frequencies of all angles was equal to 100%. The compass rose was centred on the Metaleurop Nord smelter. For each soil sampling point, the angle between the vector “Metaleurop smelter to the North” and the vector “Metaleurop smelter to the soil sampling point” was calculated and thus, for each point, the value of wind attributed corresponded to the frequency (%) of wind blowing on the point. The frequency of wind ranged from 1 to 11% (median = 7%). The prevailing wind came from the southwest and west; the higher frequencies were reported for winds coming from 260°, 240° and 200°, with frequencies of 11.1, 8.8 and 8.5%, respectively, and lower frequencies were reported for winds coming from east (100°, 120° and 140°) with values of 2.1, 1.4 and 2.7% respectively (data not shown).

The relationships between total TM concentrations in soils, distance to Metaleurop, frequency of wind and soil use were studied using general linear models (LMs). The statistical distribution of data was checked using the test of Shapiro-Wilk. Because total TM concentrations were skewed, the data were log-transformed using log10(x + 1). Because the relationship between soil concentrations and distance was not linear but logarithmic, we introduced a decimal logarithmic function in the model. The significance of the variables in the model was assessed via permutation test (Monte-Carlo, 1000 permutations), the partial R-squared (R^2^) values were calculated using an analysis of variance (ANOVA) and the pairwise comparisons were made using Tukey’s honest significant difference test. Total TM concentrations decreased with the distance from Metaleurop (p < 0.001; partial R^2^ = 0.51, 0.48 and 0.40 for Cd, Pb and Zn, respectively) and were positively related to the frequency of wind (p < 0.001; partial R^2^ = 0.08, 0.08 and 0.06 for Cd, Pb and Zn, respectively). Conditionally to distance and wind, TM concentrations differed among different soil uses (partial R^2^ = 0.11, 0.11 and 0.09 for Cd, Pb and Zn, respectively). All TM concentrations were higher in woody than in agricultural soils (p < 0.001) while they did not vary significantly between woody and urban soils (0.72 < p < 1). Pb and Zn concentrations were higher in urban soils compared to agricultural soils (p < 0.001 for Pb and Zn) although this was not the case for Cd (p = 0.12). Globally speaking, the contamination of soils ranked in the following order: dredged sediment deposit > woody ~ urban > agricultural. The R^2^ values of the models including all variables (distance, wind and soil use) reached 0.70, 0.67 and 0.54 for Cd, Pb and Zn, respectively.

Next, we developed a predictive model using the universal kriging method described by Matheron [18]. This technique allows for kriging of non-stationary data by the definition of a spatial trend. The kriging model has two components, the first one being the trend term and the second one being the spatially random variable [18,19]. The spatial trend was defined for each metal by the respective LM built above. Computation of directional variograms failed to detect a directional component. Subsequently, the spatial dependence was studied by performing omnidirectional empirical variograms with variographic envelopes (Figure S1) [19,20]. The envelops were obtained by permutation (99 simulations, Monte-Carlo method).

The models were fitted on the variograms with four correlation functions (exponential, gaussian, spherical and circular) because these functions seemed relevant due to the spatial variance pattern. Models were cross-validated (leave-one-out). The best model was chosen on the basis of the sum of squares of the fitted models and the following cross-validation criteria: mean error, mean square prediction error, mean square deviation ratio or mean square normalized error, Pearson correlation coefficient for observed versus predicted and Pearson correlation coefficient for predicted versus residuals [19,20]. For both Cd and Pb, the best models were spherical, while the best model for Zn was circular (Table S2). The parameters of the spatial functions (nugget, partial sill and range) were similar for the three metals (Table S2).

The average distance between couples of nearest points was calculated in order to choose a relevant distance between the nodes of a regular grid on which kriging would be applied. This distance was 112 m and thus, a distance of 100 m between nodes was chosen. At each node of the regular grid, the distance to Metaleurop and the frequency of wind were computed and a soil use (according to the soil use map, see “Materials and Methods”) was allocated.

Finally, we kriged soil TM concentrations at the nodes of the grid, and back-transformed obtained values (Figure 1). The goodness of fit revealed by the results of cross-validation, and the small variance of kriged values indicated that the models reliably predicted total soil TM concentrations.

## References

1. Sterckeman T, Douay F, Proix N, Fourrier H, Perdrix E (2002) Assessment of the contamination of cultivated soils by eighteen trace elements around smelters in the North of France. Water Air and Soil Pollution 135: 173-194.

2. Douay F, Pruvot C, Roussel H, Ciesielski H, Fourrier H, et al. (2008) Contamination of urban soils in an area of Northern France polluted by dust emissions of two smelters. Water Air and Soil Pollution 188: 247-260.

3. Fritsch C, Giraudoux P, Coeurdassier M, Douay F, Raoul F, et al. (2010) Spatial distribution of metals in smelter-impacted soils of woody habitats: Influence of landscape and soil properties, and risk for wildlife. Chemosphere 81: 141-155.

4. Antonic O, Legovic T (1999) Estimating the direction of an unknown air pollution source using a digital elevation model and a sample of deposition. Ecological Modelling 124: 85-95.

5. De Ridder K, Neirynck J, Mensink C (2004) Parametising forest edge deposition effective roughness lenght. Agricultural and Forest Meteorology 123: 1-11.

6. Ali-Khodja H, Boumegoura N, Habbas B (2005) Spatio-temporal deposition fluxes of dust and trace elements around a cement plant in the vicinity of Hamma Bouziane, Algeria. Fresenius Environmental Bulletin 14: 237-247.

7. Goodarzi F, Sanei H, Duncan WF (2001) Monitoring the distribution and deposition of trace elements associated with a zinc-lead smelter in the Trail area, British Columbia, Canada. Journal of Environmental Monitoring 3: 515-525.

8. Hasselbach L, Ver Hoef JM, Ford J, Neitlich P, Crecelius E, et al. (2005) Spatial patterns of cadmium and lead deposition on and adjacent to National Park Service lands in the vicinity of Red Dog Mine, Alaska. Science of the Total Environment 348: 211-230.

9. Magiera T, Zawadzki J (2007) Using of high-resolution topsoil magnetic screening for assessment of dust deposition: Comparison of forest and arable soil datasets. Environmental Monitoring and Assessment 125: 19-28.

10. Rieuwerts J, Farago M (1996) Heavy metal pollution in the vicinity of a secondary lead smelter in the Czech Republic. Applied Geochemistry 11: 17-23.

11. Skrivan P, Rusek J, Fottova D, Burian M, Minaiik L (1996) Factors affecting the content of heavy metals in bulk atmospheric precipitation, throughfall and stemflow in central Bohemia, Czech Republic. Water Air and Soil Pollution 85: 841-846.

12. Fowler D, Smith RI, Leith ID, Crossley A, Mourne RW, et al. (1998) Quantifying fine-scale variability in pollutant deposition in complex terrain using 210Pb inventories in soil. Water Air and Soil Pollution 105: 459-470.

13. Fowler D, Skiba U, Nemitz E, Choubedar F, Branford D, et al. (2004) Measuring aerosol and heavy metal deposition on urban woodland and grass using inventories of 210Pb and metal concentrations in soil. Water Air and Soil Pollution 4: 483-499.

14. Ettler V, Vanek A, Mihaljevic M, Bezdicka P (2005) Contrasting lead speciation in forest and tilled soils heavily polluted by lead metallurgy. Chemosphere 58: 1449-1459.

15. Kelly J, Thornton I, Simpson P (1996) Urban geochemistry: a study of the influence of anthropogenic activity on the heavy metal content of soils traditionally industrial and non-industrial areas of Britain. Applied Geochemistry 11: 363-370.

16. Linde M, Bengtsson H, Öborn I (2001) Concentrations and pools of heavy metals in urban soils in Stockholm, Sweden. Water Air and Soil Pollution 1: 83-101.

17. Bretzel F, Calderisi M (2006) Metal Contamination in Urban Soils of Coastal Tuscany (Italy). Environmental Monitoring and Assessment 118: 319-335.

18. Matheron G (1969) Le krigeage universel. Les Cahiers du Centre de Morphologie Mathématique, Fascicule 1. Fontainebleau, France: Ecole des Mines de Paris. 84 p.

19. Webster R, Oliver MA, editors (2001) Geostatistics for environmental scientists. Chichester, UK: John Wiley & Sons, Ltd. 271 p.

20. Bivand RS, Pebesma EJ, Gómez-Rubio V, editors (2008) Applied spatial data analysis with R. New York, USA: Springer+Business Media, LLC. 378 p.
